# AR- und Holografie-gestütztes Netzwerken als Alternative zum traditionellen Netzwerken vor Ort – ein multiperspektivischer Einblick

**DOI:** 10.1365/s40702-021-00811-2

**Published:** 2021-12-09

**Authors:** Cindy Schaefer, Aida Stelter, Anna Zeuge, Frederike Marie Oschinsky, Bjoern Niehaves

**Affiliations:** Lehrstuhl für Wirtschaftsinformatik, Universität Siegen, Kohlbettstraße 15, 57072 Siegen, Deutschland

**Keywords:** Erweiterte Realität, AR, Netzwerken, Holografie, Virtuelle Realität, VR, Augmented Reality, AR, Networking, Holography, Virtual Reality, VR

## Abstract

Virtuelles Netzwerken ist seit Beginn der Corona-Pandemie ein zentrales Mittel geworden, um sich zu vernetzen. Mit dem einschneidenden Ereignis von COVID-19 sind zahlreiche Alternativen zu traditionellen Netzwerkveranstaltungen wie Messen, Abendessen etc. entstanden. Hierbei traten jedoch auch viele Hürden auf, etwa die fehlende Menschlichkeit oder das zweidimensionale Erscheinungsbild im digitalen Raum. Ein Ansatz diese Herausforderungen zu überwinden, liegt in der Kombination von erweiterter Realität (engl. augmented reality (AR)) und Holografie. Um die technische Machbarkeit dieses Ansatzes zu überprüfen, haben wir mit AR-Experten und Kunden gesprochen und die Faktoren für und gegen die Nutzung von AR und Holografie zum Netzwerken diskutiert. Unsere Ergebnisse zeigen, dass die Experten und Kunden hohes Potenzial in der Kombination sehen. Doch mangelt es momentan noch an technologischen Lösungen, um dies breitenwirksam einsetzen zu können. Die Experten und Kunden sind sich resümierend einig, dass es, basierend auf den aktuellen technischen Entwicklungen im Bereich AR und Holografie, in naher Zukunft dafür Lösungen geben wird und die Kombination von AR und Holografie dann eine gute Alternative zum Netzwerken darstellen kann. Daraus folgern wir, dass ortsunabhängiges Netzwerken mit AR und Holografie eine Welt zum Wohlfühlen für die Nutzenden schafft und einen klaren Mehrwert darstellt.

## Abstract (en)

Virtual networking has become a key means of networking since the onset of the Corona pandemic. With this incisive event, numerous alternatives to traditional networking events such as trade fair shows, dinners, etc. have emerged. However, many barriers also arose in this process, such as the lack of humanity or the two-dimensional appearance in the digital space. One approach to overcome these barriers is the combination of augmented reality (AR) and holography. To assess the technical feasibility of this approach, we spoke with AR experts and customers and discussed the factors for and against using AR and holography for networking. Our results show that the experts and customers see high potential in the combination. However, there is currently still a lack of technological solutions to enable this to be used on a broad scale. In summary, the experts and customers agree that, based on the current technical developments in the field of AR and holography, there will be solutions for this soon and that the combination of AR and holography can then represent a good alternative to networking. From this, we conclude that location-independent networking with AR and holography creates a world of well-being for users and represents a clear added value.

## Einleitung und Motivation

Der digitale Wandel bestimmt unseren Alltag, denn „die technologischen Entwicklungen sind rasant und verändern die Art, wie wir uns informieren, wie wir kommunizieren, wie wir konsumieren – kurz: wie wir leben“ (Bundesministerium für Wirtschaft und Energie 2020). In Zeiten von Digitalisierung und der Corona-Pandemie und den damit verbundenen Kontaktbeschränkungen und Homeoffice-Reglungen rückt die digitale Zusammenarbeit zunehmend in den Vordergrund (AbuJarour et al. 2021). Zahlreiche Untersuchungen belegen die Vorteile, die mit der zunehmenden Digitalisierung einhergehen: Dadurch, dass orts- und zeitunabhängig gearbeitet werden kann, kann beispielsweise eine gesteigerte Produktivität und Effektivität erreicht, Kosten gespart (z. B. Reisekosten) und der Zugriff auf personelle Ressourcen erweitert werden (Holtbrügge und Schillo 2007). Gleichzeitig bringt die digitale Zusammenarbeit auch einige Nachteile mit sich. Ein Nachteil besteht darin, dass das Gefühl von Präsenz im Digitalen verloren oder nur schwer zu erzeugen ist (Srivastava und Chandra 2018). Ebenso ist es im digitalen Kontext deutlich herausfordernder vertrauensvolle Beziehungen aufzubauen als in Präsenz (Sarker et al. 2003). Ein weiterer Nachteil ist, dass die spontane Kommunikation entfällt oder diese nicht mehr so natürlich ist wie zuvor (Sarker et al. 2011). Konferenzen, Vorträge, Besprechungen, Diskussionen und Vernetzungstreffen bringen bislang viele Hürden der Kommunikation und Verständigung mit sich. Insbesondere das Netzwerken in Projektpartnerschaften oder mit neuen Kunden stellt sich als Herausforderung dar. Die vorhandenen Plattformen wie Skype, Zoom, MS Teams oder Webex bieten zwar initiale Möglichkeiten zum Netzwerken an, dennoch ist das Gefühl des menschlichen, ungezwungenen Miteinanders nicht dasselbe wie in Präsenz. Ein Beispiel ist das gemeinsame Mittagessen vor Ort, welches Raum für unterschiedliche Gespräche bietet und sich bislang nicht durch Videotelefonie ersetzen lässt (Deutsche Bahn 2020). Neuartige Technologien können hier zwar helfen, etablierte Strukturen neu zu denken, doch wie können wir die Möglichkeiten der Digitalisierung nutzen, sodass sich Personen von unterschiedlichen Orten und in Echtzeit in dieser Form begegnen? Virtuelle und erweiterte Realitäten (engl. virtual reality (VR) and augmented reality (AR)) in Kombination mit Holografie können hier eine Möglichkeit darstellen.

Unser Ziel ist es, mit AR und Holografie einen „Raum“ für virtuelle Treffen zu erschaffen, der die bisherigen Herausforderungen überwindet. Die Teilnehmenden sehen sich dabei als fotorealistischen Avatar oder in realer Gestalt in Originalgröße (erzeugt durch Holografie) und können mit anderen Teilnehmenden aktiv in Kontakt treten. Ein erlebbares Gemeinschaftsgefühl und eine offene, vertrauensbildende Kommunikation können wie in Präsenz entstehen. Hier fangen wir mit unserer Lösung an und zeigen, wie Netzwerken in der digitalen Welt menschlich und so real wie möglich gestaltet werden kann, damit sich Teilnehmende wohl fühlen und natürlich vernetzen können. Im Zentrum unserer Arbeit möchten wir überprüfen inwieweit die Kombination von AR und Holografie von den Teilnehmenden genutzt und akzeptiert werden, um sich zu vernetzen. Gestützt auf Werke aus dem Bereich Human-Computer Interaction und Design Science Research möchten wir herausfinden, wie die „Welt“ aufgebaut werden sollte, um sich in dieser wohlzufühlen. Wir möchten Faktoren, welche für oder gegen die neue Technologiekombination sind, identifizieren, um entsprechende Maßnahmen zur Förderung ihrer Technologieakzeptanz umzusetzen. Vor diesem Hintergrund möchten wir folgende Forschungsfrage untersuchen:

*Inwieweit wird die Kombination von AR und Holografie genutzt und akzeptiert, um sich unabhängig von Ort und Zeit mit anderen Menschen in Echtzeit zu vernetzen?*

Das vorliegende Papier ist wie folgt gegliedert: Einsteigen möchten wir in die Thematik, indem wir die Ergebnisse der ausführlichen Literatursuche zu den Themen AR, VR und Holografie, sowie die ersten Versuche von anderen Forschenden oder Unternehmen präsentieren. Anschließend erläutern wir unser methodisches Vorgehen. Im nächsten Kapitel stellen wir die Ergebnisse unserer Interviews vor. Zuletzt wird die Arbeit mit Limitationen, sowie den Implikationen für Wirtschaft und Wissenschaft abgeschlossen.

## Theoretischer Hintergrund

### Realitäts-Virtualitäts-Kontinuum

**Abb. 1:**
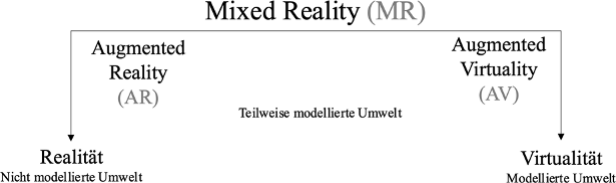
Realitäts-Virtualitätskontinuum in Anlehnung an Milgram und Kishino (1994)

Das Papier beschäftigt sich mit einer digitalen Technologie, welche eine Kombination von AR und Holografie darstellt. Um beide Begriffe zu definieren und voneinander zu unterscheiden, wird zur Einordnung des Termini das Realitäts-Virtualitäts-Kontinuum von Milgram und Kishino (1994) herangezogen (siehe Abb. 1).

Das Realitäts-Virtualitätskontinuum bildet das gesamte Spektrum zwischen den beiden Endpunkten „Realität“ und „Virtualität“ sowie die dazwischenliegenden Übergänge ab und beschreibt die gemischte Realität (engl. mixed reality oder kurz MR) als Oberbegriff (Milgram und Kishino 1994). MR umfasst dabei alle möglichen Variationen und Kombinationen von realen und virtuellen Objekten (d. h. der echten Realität und der virtuellen Realität) unter Ausschluss der beiden Extrempunkte (Hochberg et al. 2017). Kennzeichnend dabei ist der Grad der Virtualität, das heißt, ab welchem Grad der Virtualität noch von AR bzw. schon von erweiterter Virtualität (engl. augmented virtuality (AV)) gesprochen werden kann (Milgram und Kishino 1994). In der AR steht die reale Welt im Vordergrund und wird um virtuelle Elemente erweitert, zum Beispiel indem Grafiken in die reale Umgebung projiziert werden. In der AV werden in eine virtuelle Welt reale Elemente eingeblendet, zum Beispiel indem reale Objekte in Echtzeit eingeblendet werden (Mehler-Bicher et al. 2011).

### Virtuelle Realität

VR bezeichnet eine Hardware-Software-Kombination, die es dem Menschen erlaubt in computergenerierte, interaktive und dreidimensionale Räume einzutauchen sowie sich frei zu bewegen (Wexelblat 1993; Mills und Noyes 1999). Der Kern moderner VR-Hardware ist die VR-Brille mit zwei hochauflösenden Displays zur Darstellung künstlich erzeugter Bilder (Burdea und Coiffet 2003; Dörner et al. 2019). Die VR-Brille ist an eine Sensorik gekoppelt, die die Lage und Position des Kopfes erfasst. Bewegt die nutzende Person den Kopf, registriert die Sensorik die Veränderung von Lage und Position und passt die erzeugten Bilder an (Wexelblat 1993; Tißler 2018). Vergehen zwischen dem Sensorik-Signal und der Bilderzeugung weniger als elf Millisekunden (Motion-to-Photon-Latency), so entsteht der Eindruck der virtuellen Realität (Wexelblat 1993; Dörner et al. 2019; Wohlgenannt et al. 2020). Mithilfe von Controllern kann die benutzende Person zudem mit Objekten interagieren und sie manipulieren (Martín-Gutiérrez et al. 2017).

VR-Technologie charakterisiert sich durch Immersion, Präsenz und Interaktivität (Walsh und Pawlowski 2002; Wohlgenannt et al. 2020). *Immersion* charakterisiert sich durch die subjektive Erfahrung sich vollständig im virtuellen Raum involviert zu fühlen und ermöglicht es der nutzenden Person, sich innerhalb des virtuellen Raums zu bewegen und mit anderen Personen und Objekten zu interagieren (Wexelblat 1993; Suh und Lee 2005; Dede et al. 2017). *Präsenz* beschreibt das subjektive Gefühl, ob die nutzende Person die virtuelle Umgebung als reale Welt nachempfinden kann (Sanchez-Vives und Slater 2005). Das Gefühl der Präsenz lässt sich nach Lee (2004) in drei Komponenten untergliedern: räumliche Präsenz (Werden die Objekte in der virtuellen Welt als real wahrgenommen?), soziale Präsenz (Werden die anderen Personen als real wahrgenommen?) und Selbstpräsenz (Wird die eigene Präsenz im virtuellen Raum als real wahrgenommen?). *Interaktivität* beschreibt das Ausmaß, in dem die nutzende Person Einfluss auf die Form oder den virtuellen Raum nehmen kann (Steuer 1992).

### Erweiterte Realität

AR ermöglicht den Nutzenden in der eigenen Umgebung zu bleiben und diese, um 3D registrierte Gegenstände oder Charakteristika, zu *ergänzen* (Azuma 1997; Amin und Govilkar 2015). Damit wird die reale und die virtuelle Welt *kombiniert*. Wichtig ist, dass die Ergänzungen in *Echtzeit* erfolgen, um die *Interaktivität* mit der realen Umgebung zu gewährleisten. Diese drei Eigenschaften bilden auch gleichzeitig die drei Bedingungen an AR-Systeme (kurz ARS) ab, die Azuma im Jahre 1997 postulierte (Azuma 1997). Die Nutzung von AR hat im Gegensatz zur Nutzung von VR den Vorteil, dass die Nutzenden nicht vollkommen von ihrer Umwelt abgeschottet sind und die Umgebung nicht rein virtuell dargestellt, sondern mit zusätzlichen Informationen erweitert wird (Deutsche Telekom 2020).

Für die Anwendung von ARS werden verschiedene Komponenten benötigt. Zum einen die Hardware, die die 3D registrierten Elemente überträgt. Hier gibt es für unseren Fall drei relevante Formen. Erstens, feste Displays, die ein 3D registriertes Objekt in den Raum erzeugen. Zweitens, sogenannte handheld Displays, also Smartphone- oder Tablet-Displays, mit denen die Objekte an den gewünschten Orten sichtbar werden. Und drittens eine AR-Brille oder ein so genannter Head-Mounted-Display (kurz HMD), welche mit Sensoren und Kameras ausgestattet ist. Die beiden letzten Arten nennt man auch mobile System, da hier eine uneingeschränkte Bewegungsfreiheit gewährleistet ist. Für die Positionierung der Gegenstände ist wichtig, dass die Position und die Richtung zu jeder Zeit definiert sind, um Schwimmeffekte zu vermeiden. Als Schwimmeffekte werden die Zeiträume bezeichnet, wenn das virtuelle Bild der realen Umgebung hinterherhängt und somit eine Differenz sichtbar wird. Zum anderen werden die 3D-Objekte benötigt, um sie darzustellen. Dafür müssen die 3D-Objekte in den zugehörigen Cloud Systemen programmiert und abgespeichert worden sein. Für die Echtzeit Anwendung werden abschließend eine ausreichende Übertragungskapazität und Download-Bandbreite benötigt (Azuma 1997; Tönnis 2010; Wursthorn 2019).

### Holografie

Holografie ist eine Zusammensetzung der beiden griechischen Worte „holos“ („vollständig“) und „graphein“ („Aufzeichnung“). Es handelt sich um ein Verfahren, welches die Lichtwellen eines Objektes in mehreren Dimensionen aufnimmt (Fränzl et al. 2013). Der Unterschied zur Fotografie liegt darin, dass bei der Fotografie ein zweidimensionales Bild eines Objektes dargestellt wird, während die Holografie das gestreute Wellenfeld eines Objektes untersucht und ein dreidimensionales Bild darstellt (Ostrowski und Osten 2013; Voss-de Haan 2020). Dabei wird eine Aufnahme eines körperlichen Objektes als ein dreidimensionales Bild in einem realen Raum wiedergegeben, welches realitätsnah dargestellt wird (Bendel 2021).

Das Hologramm bzw. das dreidimensionale Abbild eines Objektes ist eine „kohärente, monochromatische Welle“ (Voss-de Haan 2021), die in zwei Wellen (Objekt- und Referenzwelle) aufgeteilt wird und durch eine Strahlungsquelle, in der Regel mit einem Laser, erzeugt wird. Die Objektwelle wird von einem Objekt gestreut und mit einer ungestreuten Referenzwelle zur Interferenz gebracht, welche Informationen beinhaltet und dementsprechend ein Interferenzmuster abbildet. Durch die Beleuchtung dieser mit einer entsprechenden identischen Welle, wird das Inferenzmuster zu dem ursprünglichen Wellenfeld abgebildet und das Hologramm erscheint als ein virtuelles 3D-Abbild eines Objektes. Durch die Wiederherstellung des aufgenommenen Wellenfeldes, kann im Unterschied zu einer Fotografie das Objekt anschließend aus unterschiedlichen Richtungen betrachtet werden (Voss-de Haan 2021).

### Umsetzung

#### Technische Umsetzung

AR ist die Grundlage für eine funktionierende Holografie-Anwendung. Hierfür sind zwei unterschiedliche Komponenten nötig: eine AR-Kollaborationsplattform und eine MR-Brille. Eine AR-Kollaborationsplattform muss die Möglichkeit bieten, ein 3D-Modell in eine Cloud hochzuladen und dann mit Hilfe eines AR-Devices wieder anzuschauen. Das zu holographierende Objekt muss aus verschiedenen Perspektiven abgescannt werden und danach ohne technische Verzögerung, sogenannter Latenz, an einem anderen Ort wieder zu sehen sein. Eine Herausforderung im Bereich der Holografie ist vor allem, dass die Visualisierung der Hologramme am Zielort möglichst ohne technisches Zusatzequipment stattfinden sollte, insbesondere wenn diese für die menschliche Kommunikation eingesetzt werden soll (Voss-de Haan 2021).

Die MR-Brille, welche AR umsetzt, muss die Funktion haben, dass sich mit Hilfe von Sprache und Gesten die AR-Anwendungen steuern lassen. Die MR-Brille blendet dabei eine 3D-Projektion in eine natürliche Umgebung innerhalb der realen Welt ein. Dadurch kann die Realität durch beispielsweise Grafiken, Texte, 3D-Modellen oder Informationsboxen erweitert werden. Die Einsatzmöglichkeiten sind dabei sehr vielfältig. Es können Prozesse effizienter gestaltet und Simulationen durchgeführt werden, um Objekte, die in der Realität noch nicht existieren, zu testen. Des Weiteren lassen sich Produkte und Services unabhängig vom Ort in Echtzeit vorstellen und den aktuellen Gegebenheiten und Anforderungen anpassen (Luber und Litzel 2018; Microsoft Corporation 2020).

#### Praktische Ausgangssituation

VR-Anwendungen versetzen den Nutzenden in eine simulierte 3D-Umgebung. Durch die Verwendung von VR-Brillen werden Videos und Bilder in 3D-Format gezeigt. Die Bildausschnitte passen sich den Augen- und Kopfbewegungen der Nutzenden an und ermöglichen in Kombination mit Bewegungssensoren die Erkundung von 3D-Welten (Anthes et al. 2016). Die Einsatzmöglichkeiten für VR reichen von Videospielen über das Bereisen von Orten, bis hin zur Bildung und Gesundheitsanwendungen. Das Thema AR ist spätestens seit dem Durchbruch von Spielen wie Pokémon Go populär und die Potentialentfaltung befindet sich auf dem Vormarsch. Für die Entwicklung von AR ergeben sich aufgrund der standortunabhängigen Nutzungsmöglichkeit sowie der einfachen Integrationsmöglichkeiten in den Alltag der Konsumenten zahlreiche Anwendungsfelder.

So wie Smartphones und Tablets es bereits heute ermöglichen, zu jedem Zeitpunkt auf Informationen und nützliche Funktionen zurückzugreifen, könnte durch die direkte Einbindung von virtuellen Elementen in die reale Umgebung eine beschleunigte und intuitive Informationsaufnahme erfolgen (auch im Bereich der Kommunikation). Marktstudien zeigen, dass derzeit von einem Marktpotenzial hinsichtlich der Entwicklung von VR und AR ausgegangen werden kann (Statista 2020). Auch in Deutschland wird ein hohes Marktpotential vorausgesagt (Hochrechnungen für 2020 schätzen einen Umsatz von über 800 Mio. €) (Statista GmbH 2020), allerdings sind gerade im B2C-Sektor noch wenige Anwendungen marktreif. So bieten zwar die beiden herrschenden mobilen Betriebssysteme Android (Google) und iOS (Apple) für Entwickelnde sogenannte Software Development Kits an, bisher ist jedoch eher eine kleine Anzahl von AR-Apps veröffentlicht (Apple 2020).

## Methodisches Vorgehen

### Methodenwahl und Datensammlung

In unserem Papier haben wir einen qualitativen Ansatz (Flick et al. 2004) verwendet, um zu erforschen inwieweit neue Technologien, vor allem die Kombination von AR und Holografie, von den Teilnehmenden genutzt und akzeptiert werden, um sich zu vernetzen. Wir sind dabei deduktiv vorgegangen, indem wir sowohl die Experten- als auch die Kunden-Perspektive betrachtet haben, um Schlussfolgerungen zu ziehen.

Im Rahmen unseres Papiers haben wir virtuelle Interviews mit einer durchschnittlichen Länge von 60 min durchgeführt. Die Interviews wurden im Juni, Juli und Oktober 2021 in Deutschland durchgeführt. In Tab. 1 sind die Informationen zu den Interviewteilnehmenden angegeben, wobei wir zwischen Experten und Kunden differenziert haben. Wir befragten also Mitarbeitende von Unternehmen, die sich alltäglich mit VR, AR und MR beschäftigen, jedoch in der Größe (d. h. kleine und mittlere Unternehmen und Start-ups) variieren. Diese Verteilung über die Hierarchieebenen war zufällig, kann aber in Kombination mit der unterschiedlichen Anzahl an Berufsjahren sicherstellen, dass individuelle und elitäre Verzerrungen vermieden und unterschiedliche Perspektiven berücksichtigt werden (Miles und Huberman 1994).

**Tab. 1:** Demographische Angaben der Interviewpartner

Nr	Interviewteilnehmer	Geschlecht	Alter	Ausbildung	Beruf
1	Experte (EX1)	M	38	Studium	Gründer und Geschäftsführer eines Start-ups, dass sich mit skalierbaren AR/MR-Anwendungen für die Industrie beschäftigt
2	Experte (EX2)	W	36	Studium	Technische Projektleiterin in einem mittelständischen Unternehmen
3	Experte (EX3)	M	30	Studium	IT-Stabstellenleiter u. a. verantwortlich für die Einführung von VR und AR Anwendungen im Unternehmen
4	Kunde (KU1)	M	54	Promotion	Betriebswirt
5	Kunde (KU2)	M	28	Studium	Banker
6	Kunde (KU3)	M	40	Studium	Berater für Kommunen
7	Kunde (KU4)	W	28	Studium	Verkehrsplanerin
8	Kunde (KU5)	M	39	Studium	CEO eines Mobilitätsdienstleister
–	–	Im Durchschnitt	36,6	–	–

Um ein breiteres Spektrum an Antworten zu erhalten und den Befragten zu ermöglichen, frei und offen zu sprechen, verwendeten wir einen halbstrukturierten Leitfaden mit offenen Fragen (Pumplun et al. 2019). Wir folgten Sarkers Leitfaden für qualitative Forschung, um typische Fallstricke qualitativer halbstrukturierter Interviews zu vermeiden (Sarker et al. 2013; Pumplun et al. 2019).

Der Interviewleitfaden ist in vier Kategorien unterteilt. Die erste Kategorie umfasst Fragen zum Befragten (z. B. Alter, Hintergrund, IT-/VR-/AR-Kompetenz). Die zweite Kategorie beschäftigt sich mit Netzwerken im Allgemeinen, beispielsweise „Wie haben Sie sich vor COVID-19 vernetzt? Wie sind Sie auf Partnerakquise gegangen? Wie hat sich dies durch die Pandemie verändert?“. Die dritte Kategorie fokussiert das Netzwerken mit Hilfe von VR, AR und Holografie. Beispielhafte Fragen sind hier „Wie umfangreich hat virtuelles Netzwerken vor COVID-19 stattgefunden? Welche Technologien (VR, AR und/oder Holografie) haben Sie vor COVID-19 schon mal zum virtuellen Netzwerken genutzt? Und wenn ja, wie haben Sie dies empfunden?“. Danach haben wir einige Fragen zum Netzwerken nach COVID-19 gestellt, um Veränderungen mit einzubeziehen, z. B. „Was hat sich durch COVID-19 verändert? Welche Technologien (VR, AR und/oder Holografie) haben Sie im Laufe der COVID-19 Zeit zum virtuellen Netzwerken ausprobiert? Wer ist Treiber dieser Veränderung?“. In der vierten Kategorie stellten wir schließlich Fragen zu den Vor- und Nachteilen von VR, AR und/oder Holografie, im Gegensatz zum traditionellen Netzwerken, z. B. „Welche Vor- und Nachteile sehen Sie bei der Nutzung und Umsetzung von VR, AR und/oder Holografie zum virtuellen Netzwerken?“.

### Datenanalyse

Die Interviews wurden aufgezeichnet, nonverbal transkribiert und mit der Software MAXQDA (VERBI Software. Consult. Sozialforschung GmbH 2021) ausgewertet. Für die Analyse der Interviews wurde Bottom-up Coding verwendet. Zwei Forschende wendeten unabhängig voneinander das offene Kodieren an, d. h. den Sätzen und Absätzen wurden Code-Phrasen zugeordnet, die den Inhalt am besten repräsentieren (Corbin und Strauss 2014; Glaser und Strauss 2017). Anschließend haben wir die Ergebnisse verglichen und gruppiert (axiales Kodieren), um akzeptanz- und nutzungsspezifische Aspekte zu finden (Corbin und Strauss 2014). Unterschiedliche Meinungen wurden mit einem dritten Forschenden diskutiert und einvernehmlich geklärt. Zum Beispiel bei dem folgenden Zitat: „Also sprich ich bin an dem einen Tag in Berlin und dann klappere ich mal einfach alle Kunden ab. Und wenn es einfach nur eine halbe Stunde ist. Ich schau einfach mal vorbei und guck, was sich Neues ergibt bei den Bestandskunden.“ (EX2), wurden zwei unabhängige Codes („Kundenkontakt“ und „neue Kontakte treffen“) gefunden. Schließlich wurde „Kundenkontakt“ als axialer Code verwendet. Anschließend wurden die axialen Codes nach Aspekten gruppiert. Wir beendeten die Analyse mit Sättigung, d. h. wenn keine neuen übergeordneten Aspekte gefunden wurden.

## Ergebnisse

Netzwerken hat eine große Bedeutung für Menschen. Der Austausch, die Diskussion und gemeinsame Kommunikation mit anderen Menschen gehören sowohl im privaten als auch im beruflichen dazu. Die Vernetzung mit anderen Menschen ist essenziell und von großer Relevanz, wobei es viele verschiedene Arten und Wege zum Netzwerken gibt. Sei es per Telefon, in Präsenz vor Ort z. B. bei Messen, Veranstaltungen, etc. oder auch das virtuelle Netzwerken. Die Experten haben hierbei unterschiedliche Ansichten. EX1 ist ganz klar der Ansicht, dass virtuelles Netzwerken ein Generationsthema ist.

*„[…] [W]eil wir alle IT verstehen, und noch zu der jüngeren Generation gehören, ist es einfacher. Aber wenn ich mir jetzt angucke – das ist übrigens ein Generationsthema […] – ich habe eine Mitarbeiterin, die macht für uns Social-Media-Kommunikation, die ist ganz anders unterwegs, als ich es jetzt bin und wir sind nur 15 Jahre auseinander. […] Da ist schon ein deutlicher Unterschied und damit steht und fällt natürlich auch die Art und Weise, wie ich darüber kommuniziere und ob ich bereit und offen bin.“* (EX1)

Anderer Meinung sind jedoch die Experten 2 und 3. Sie verdeutlichen, dass die Art und Weise wie man kommuniziert und netzwerkt nicht vom Alter, sondern von der Persönlichkeit und Technikaffinität abhängt und nach Belieben gewählt und genutzt wird.

*„Leute, die technisch affin sind und Spaß an sowas haben. […] [D]as ist glaub ich zumindest teils altersunabhängig. […] [I]m Kollegenkreis […] ist einer meiner ältesten Kollegen derjenige, der am meisten Spaß dran hat, weil er einfach vom Typ her so ist. Ganz generell würde man vermuten, dass die jüngeren Leute, weil sie mit Gaming, Smartphone und so weiter groß geworden sind, dann noch mehr Interesse haben. Ich glaube sie bringen einfach noch mehr Basiswissen mit und finden sich einfach schneller in grundsätzlichen Funktionsweisen ein. Das heißt aber noch lange nicht, dass diejenigen mehr Interesse haben an neuen Technologien. Das ist schon ein Persönlichkeits-Thema.“* (EX2)

*„Wir führen gerade eine Studie durch, wo wir fast jeden Mitarbeiter […] mal in die VR oder AR gesteckt haben. Ich finde sehr spannend, dass es so einen Altersgap in meinen Augen nicht gibt. Es gibt glaube ich eher ein Technikgap, also ob einer so ein bisschen neugierig auf neue Technik ist. Also das Alter alleine ist glaube ich keine Größe, die das limitiert.“* (EX3)

### Netzwerken vor der COVID-19 Pandemie

Netzwerken hat auch vor der COVID-19 Pandemie eine entscheidende Rolle eingenommen. Die Art und Weise wie kommuniziert wurde war eine andere, mehr in Präsenz und von Angesicht zu Angesicht.

*„Vor Corona ging das relativ gut. Man konnte zu irgendwelchen Veranstaltungen fahren und irgendwelche Leute treffen. Das funktioniert aber heute leider nicht mehr.“* (EX1)

*„Rein beruflich gesehen waren viele Kundenworkshops vor Ort. […] Also normalerweise waren gerade diese Kick-off Phasen im Projekt vor Ort.“* (EX2)

*„Vor COVID […] war gerade der Austausch rechts, links, davor oder danach von Workshops […] sehr gewinnbringend. Man hat sich bei einem Kaffee oder einer Cola […] einfach zusammengesetzt und hat über [den Workshop] gesprochen […]. Und man war relativ schnell durch Smalltalk in einem sehr guten Netzwerk unterwegs oder hat sich seine Kontakte gebildet. Dann tauscht man irgendwann Visitenkarten aus […] und dann war das ein Selbstläufer. Also das ist häufig so bei vor-Ort Veranstaltungen.“* (EX3)

Bestehende, aber auch neue Kunden konnten so an einem Tag direkt vor Ort besucht werden, um sich auszutauschen, an möglichen Problemen direkt zu arbeiten und zu zeigen, dass sich jemand kümmert. Diese Netzwerkpflege wurde vor der COVID-19 Pandemie meist in Präsenz durchgeführt.

*„Ich bin an dem einen Tag in Berlin und dann klappere ich mal alle Kunden ab und wenn es einfach nur eine halbe Stunde ist. Ich schau einfach mal vorbei und guck, was sich Neues ergibt bei den Bestandskunden.“* (EX2)

*„Wir wurden im Sommer immer von unseren Verkäufern zum Sommerfest eingeladen. Es gab Essen und man konnte in entspannter Atmosphäre sprechen. Da haben wir sogar unseren letzten großen Deal abgeschlossen.“* (KU5)

Virtuelles Netzwerken war auch vor der COVID-19 Pandemie präsent, jedoch wurden die Möglichkeiten der digitalen Technologien nur wenig bis gar nicht umgesetzt und genutzt. Viele Veranstaltungen und Meetings wurden in Präsenz vor Ort und kurze Absprachen per Telefon durchgeführt. Videotelefonie wurde gelegentlich als Ausgleich zum Telefonieren genutzt. Die Technologie gab bereits, diese wurde jedoch nicht im vollen Ausmaß genutzt.

*„Also was da mal passiert ist, ist dass Du mal mit einem Kunden einen Videocall hattest. Das war aber dann auch schon das Höchste der Gefühle. So nach dem Motto, wir müssen uns ja jetzt nicht treffen, lass uns doch einen Webex Call oder so machen, anstatt zu telefonieren. Aber schlussendlich gab es das nicht. Es gab keine virtuellen Veranstaltungen.“* (EX1)

*„Vor COVID hat es anders stattgefunden, wenn es schon virtuelle Termine gab [als heute]. […] All diese Kommunikation in der ersten Runde hat meistens virtuell stattgefunden. Spannend sogar ohne Kamera – alle haben nur so ein bisschen gequatscht, so Telefonkonferenz-mäßig.“* (EX3)

Weiter haben die Interviewpartner verstärkt darauf hingewiesen, dass die Hürden des virtuellen Netzwerkens vor der COVID-19 Pandemie insbesondere daran lagen, dass Menschen sich gerne vor Ort mit anderen Menschen unterhalten wollten. Es ist zum einen einfacher, da keine weiteren Gegenstände bzw. Fähigkeiten benötigt wurden und zum anderen haben sie von einem anderen Gefühl gesprochen. Das Gefühl mit echten Menschen zu reden, sie zu sehen, ihre Gestik und Mimik richtig deuten zu können und unbeschwert ohne vorherige Zeitabstimmung sich unterhalten zu können.

*„Menschen sind persönlich. Menschen wollen Menschen treffen. Einfach das echte Miteinander und deswegen wollen die Menschen halt auch Veranstaltungen und deswegen sind Messen so groß, weil da kann ich viele Leute treffen. Ich kann ein bisschen quatschen und dabei auch versuchen Business zumachen. Durch Corona ging es nicht mehr – alle mussten umlernen.“* (EX1)

*„Sich Treffen ist immer noch etwas anderes als digital, also wenn man mit der Person dann tatsächlich an der Ecke steht, am Tresen steht, am Tisch sitzt und Miteinander isst oder sowas. Das sind andere Emotionen.“* (KU3)

*„Wenn ich virtuell das mache, was auch möglich wäre, so rein vom Informationsgehalt her, fällt aber dieser informelle Austausch massiv weg und das ist für mich der eigentliche Punkt vom Netzwerken oder Beziehungsaufbau, um dann eigentlich erst über das Fachliche und gemeinsame Projekte zu sprechen.“* (EX2)

### Netzwerken während der COVID-19 Pandemie

Durch die plötzliche Distanz, bedingt durch die COVID-19 Pandemie, wurde persönliches und gemeinsames Arbeiten und Netzwerken unmöglich und neue Methoden des virtuellen Netzwerkens wurden benötigt, die insbesondere durch den digitalen Wandel immer mehr an Zuspruch gewonnen haben. Zunächst wurde auf Vorhandenes zurückgegriffen, wie Telefonate, Videobesprechungen und Social-Media wie LinkedIn und XING.

*„Ich habe Instagram und auch von der Firma haben wir alle [Social-Media] Kanäle eigentlich. […] Aber das Meiste kommt über LinkedIn.“* (EX1)

*„Grundsätzlich [nutze ich Social-Media-Accounts] ja. Wenn ich Kontakt mit jemandem hatte, über LinkedIn oder XING, gerade im beruflichen Kontext, schreibe ich teils über diese Kanäle die Leute noch an.“* (EX2)

*„Das hat in der Tat zugenommen. Also Social Media, XING, LinkedIn nutze ich für berufliche Zwecke, um mich zu verlinken und mit Menschen in Kontakt zu treten. Also LinkedIn, XING hat beruflich extrem zugenommen. LinkedIn sogar noch mehr als XING. [..] Das habe ich vor Covid gar nicht so auf dem Schirm gehabt, wenn ich ehrlich bin.“* (EX3)

Dabei wurde jedoch schnell deutlich, dass es in naher Zukunft keine Alternative geben wird und dass Alternativen für Veranstaltungen, Messen etc. gefunden werden müssen. Hier wurden durch die Experten initiale Lösungen, wie Erkundungstage, BarCamps oder Kaffeerunden beschrieben und auch das Gefühl des Nutzungszwangs wurde deutlich.

*„Wir haben viele Veranstaltungen […] und das ist eigentlich auch nach wie vor so, bloß dass wir es einfach digital machen. Also diese Discovery Days und dergleichen finden jetzt einfach kürzer und digital statt. Das geht auch online, aber […] für mich persönlich, ich muss mich dazu zwingen. Es gibt bei vielen solcher Veranstaltungen inzwischen so Kaffee-Talks oder sowas. Das heißt, Du gehst aus dieser Session raus und wählst dich in eine Neue ein, um dann quasi informell zu sprechen. Das fühlt sich so gezwungen an. [..] Das finde ich schwierig.“* (EX2)

*„Wir hatten jetzt vorletzte Woche einen Vortrag bzw. eine Tagung in der virtuellen Realität mit virtuellen Avataren. Also Du bist wie bei einem Computerspiel da durchgelaufen und hattest deinen Avatar und konntest dann mit den Leuten in deiner Umgebung reden. Du hattest auch einen menschlichen Avatar, also das war jetzt keine Pixelfigur, sondern das sah schon alles sehr realitätsnah aus. So bei 75 % von der Realität […].“* (EX3)

Diese initialen Lösungen stellen jedoch keinen adäquaten Ersatz für Netzwerkveranstaltungen in Präsenz dar, weshalb wir mit den Experten und Kunden innovative Lösungen diskutiert haben. Die Interviewteilnehmer bedauern jedoch, dass es bis heute keine zufriedenstellende Lösung gibt.

*„Ich mag zum Netzwerken sehr gerne wonder.me. Ich finde Konferenzen dann nicht effektiv, wenn sie komplett geschlossen sind, sprich ich kann nicht mal sehen, welche Teilnehmer da sind. Dann ist das wie ein Film, den ich mir angucke, aber ich habe keinen Netzwerk-Effekt.“* (EX1)

*„Ja also, wir hatten eine Konferenz. Eine Fachkonferenz mit diversen Vorträgen und auch Räumen, wo Detailthemen dann näher beleuchtet worden sind, Workshops hatten und die Konferenz war vorbei. Und man konnte sich später in einem virtuellen Raum treffen. Man hatte so ein Avatar, mit dem man sich dann durch den Raum bewegen und sich zu anderen Personen dazu stellen konnte. Also technisch sicherlich eine echt gute Leistung und auch beeindruckend irgendwie. Aber ich bin in dem Sinne nicht der Netzwerker, der auf jemanden zugeht und sagt „Hallo, ich bin der und der, arbeitete für den und den, und ich möchte mich jetzt mit dir vernetzen.“ Bei mir entstehen Netzwerke irgendwie anders. Na also, informeller und auch klar, irgendwo durch Sympathie geprägt und nicht wenn jemand sagt, Du musst jetzt da auf der Konferenz zu dem und dem gehen. Dann funktioniert das Netzwerken zu 90 % nicht.“* (KU3)

Erste Ansätze für Netzwerkveranstaltungen mit AR empfinden die Experten als spannend und bereichernd, da somit fehlende Aspekte des Netzwerkens, wie das Gefühl des Raumes, der Nähe und der ungezwungene, spontane Kontakt möglich werden. Hierbei sind jedoch auch die Hürden des AR-Netzwerkens deutlich geworden. Es beginnt bei den digitalen und technischen Kompetenzen der Teilnehmenden und endet beim aktuellen Stand der technischen Umsetzbarkeit.

*„AR ist erklärungsbedürftig. Du musst zu den Leuten hin, Du musst denen Brillen aufsetzen, Du musst denen was zeigen und das funktioniert nicht über die Distanz.“* (EX1)

*„Ich war teils nur überrascht […] wie unterschiedlich die [digitalen] Kompetenzen sind. Also für mich war das keine Umstellung, weil ich habe davor schon viele Online-Meetings gehabt und auch viel mit den Microsoft Produkten gearbeitet usw. Aber es gibt auch Abteilungen […], die kannten das so gar nicht.“* (EX2)

*„Wenn alle aus dem Team so eine Brille hätten und wirklich teilnehmen könnten und man nicht dann aufgrund von Technologiemangel jemanden ausgrenzt, da könne ich mir das schon ganz gut vorstellen. Vor allen Dingen, wenn es ums Netzwerken geht, dass man sich einfach vor den Veranstaltungen noch trifft und den Austausch hat. Aber so diese Firmenevents mit einem Grillfest oder sowas, da könnte ich mir schlecht vorstellen, solche Events [über VR oder AR] zu machen. Aber so ein Austauschtreffen kann ich mir gut vorstellen.“* (KU4)

*„Ich glaube, wenn man sich in so einer virtuellen oder erweiterten Realität befindet, mit einer Brille auf und nichts um sich Drumherum mitbekommt, weil man auch mit Ton arbeitet, mit anderen Personen spricht, dann sollte man sich auch sicher sein, dass jetzt nicht irgendwann einer reinkommt und einen beobachtet zum Beispiel. Das könnte für viele auch unangenehm sein.“* (KU2)

*„Eine AR-Veranstaltung hat noch nicht stattgefunden, weil es technologisch extrem schwierig ist und noch gar nicht so funktioniert tatsächlich. Es gibt zwar einzelne Möglichkeiten, wie Du das machen kannst, aber noch nicht so stabil, dass Du das hinkriegst. Wenn ich einen Menschen in 3D erzeugen möchte […] ist das Problem, dass Du den Menschen von allen Seiten abscannen musst, und zwar dauerhaft, um ihn dann auch wieder so zusammen zu setzen. Denn es bringt ja nichts, wenn ich [eine Person] von vorne 3D scanne und wenn ich dann aufstehe, ist [die Person] von hinten flach.“* (EX1)

Netzwerken lebt nun mal von vielen Menschen an einem Ort und hier erlangt die technische Umsetzbarkeit heute ihre absolute Grenze. Möchte man dies um Echtzeit und mit sowohl holografischer Gestik als auch Mimik kombinieren, ist dies heutzutage nicht möglich. Auch ist das Gewicht der Brillen auf Dauer zu hoch.

*„Mit einer Person mag das noch funktionieren. Wenn ich jetzt hier im Raum vier Kameras aufhänge, dann kriege ich das schon irgendwie hin, mich abzuscannen und mich woanders hin zu positionieren und ich weiß, dass der andere sich das auch angucken kann. Aber wenn Du nun das mit einer Gruppe von Personen machen willst, die in einem Raum sind, stößt Du an technische Grenzen. Daher hast Du alleine schon so technische Herausforderungen wie, was mache ich, wenn der eine Sensorstrahl, den einen Menschen gar nicht erwischt, weil der durch einen anderen Menschen versteckt wird.“* (EX1)

*„Was ich als einen Hauptpunkt sehe, ist ein bisschen auch Kompetenz. Also so eine VR/AR-Brille entsprechend zu nutzen, bedarf ein bisschen technisches Geschick. Bei VR auch entsprechend viel Platz. Das sind limitierende Größen.“* (EX3)

Zuletzt hängt der Erfolg einer solchen Netzwerkveranstaltung von der Akzeptanz der Teilnehmenden ab. Selbst wenn alle technischen Probleme beseitigt wären und auch bei allen Teilnehmenden die Kompetenzen und das Equipment für AR und Holografie Netzwerkveranstaltungen vorhanden wäre, müsste es genutzt werden. Daher stellt die Akzeptanz einen wichtigen Faktor dar. In den Interviews haben wir jedoch auch erfahren, dass die Altersklasse bei der Akzeptanz eine untergeordnete Rolle spielt. Viel entscheidender ist, ob hybride Arbeit vor der COVID-19 Pandemie schon gelebt wurde. Denn in diesem Fall besteht laut den Experten eine höhere Akzeptanz für innovative Methoden, als wenn zuvor alles in Präsenz stattgefunden hat.

*„Ich finde es erstaunlich, wie stark wir es unterschätzt haben, wie lange es dauert, bis alles Akzeptanz findet und es auch wirklich funktioniert. Natürlich gibt es immer [einen Prozentsatz], die vorrennen und wo es funktioniert. […] Aber es gibt auch die große Masse, die dann erst nachzieht. Selbst wenn ich das technologisch beherrschen würde, muss meine Führungskraft das ja auch noch wollen und auch noch akzeptieren. […] Aber ich glaub schon, dass die Akzeptanz nach oben gerutscht ist […], weil auch die Formate besser geworden sind.“* (EX1)

*„Also fast alles ist irgendwie technisch machbar, aber es wird halt nicht richtig genutzt. Also ob wir jetzt wirklich mangelnde Kompetenz oder mangelnde Regeln drum herum oder auch Prozesse oder auch der Mut fehlt, sich mit Dingen mal auseinanderzusetzen, ist nicht entscheidend. Es muss genutzt werden.“* (EX2)

*„Akzeptanz ist ein ganz großes Thema und eine entsprechende Heranführung für alle Altersgruppen und alle Wissensstände ist hierbei notwendig. Also, in der kommunalen Verwaltung ist es ja so, dass der Großteil der Menschen, die dort arbeiten, zwischen, ich schätze mal, 45 und 56 sind. Entsprechend hängen die IT-Entwicklung und Akzeptanz hinterher. Wenn ich jetzt meine Kollegin angucke, die auch mit den ganzen neuen Dingen wie TikTok und wie sie alle heißen vertraut ist – bei mir hat es irgendwo bei WhatsApp und Facebook aufgehört. Heute mache ich das und das; ich habe ein komplett diverses Feld an Wissen und auch an Akzeptanz entwickelt und da muss ich schon darauf achten, dass ich meine Begeisterung beibehalte und auch die Akzeptanz nochmals Neues zu lernen aufrechterhalte und mich selbst motiviere. Vielleicht ist es auch dann eher was, wo man sagt, neue Technologien, fangen wir mit den Jüngeren an, weil dann kann ich so etwas auch schneller einsetzen und die älteren Menschen oder nicht so bereitwilligen Menschen, lernen dies später.“* (KU3)

### Netzwerken nach der COVID-19 Pandemie – ein Ausblick

Netzwerken bleibt nach all den Veränderungen durch die COVID-19 Pandemie Teil der beruflichen DNA. Nur durch ein effektives Netzwerk können neue Kunden akquiriert werden und Bestandskunden gehalten werden. Die Experten denken, dass eine Veränderung in der Welt des Netzwerkens entstehen wird. Eine Hypothese ist, dass die beiden Teile einer klassischen Veranstaltung (inhaltliche Präsentation und anschließendes Netzwerken) getrennt werden.

*„[Ich glaube,] dass es keine Großveranstaltungen mehr geben wird. […] Also eher wird es kleineren Netzwerk-Events geben, wo Du so 50 Leute zusammenbringst als 50.000.“* (EX1)

*„Ich finde, dass man eine klarere Trennung hat zwischen dem fachlichen, relevanten Teil. Dass man den halt einfach konzentrierter rüberbringt und dann ganz bewusst, aber dann auch mehr Zeit für Informelles hat. Und dann ist es aber auch die informelle Zeit. […] Denn ich denke mir, den Inhalt hätte ich mir auch einfach auf einer Seite durchlesen können oder fünf Minuten YouTube Video anschauen oder was auch immer. Das heißt dafür muss ich nicht vor Ort sein. Wiederum für den informellen Teil, einfach austauschen, auf Ideen kommen, so diese kurzen Impulse auch in Gesprächen aufzuschnappen, Menschen kennenzulernen – das ist für mich schon eher noch in Persona vor Ort.“* (EX2)

*„Kontakte hier aus der Region, mit denen man auch in Zukunft noch sprechen möchte oder wo der Kontakt einem was bringt, sind wichtig für Präsenz. Digital gerne da, wo ich 1–2 h hinzufahren müsste und der Inhalt nur mäßig relevant ist, im Zweifel nehme ich dann auch in Kauf einen Tick weniger zu Netzwerken. Aber die Veranstaltungen, die mir am Herzen liegen, wo ich mir vom Netzwerken etwas […] da möchte ich hinfahren, weil da sind Firmen hier aus der Region, […] da geht es hin, da will ich mit den Leuten reden, die möchte ich Face-to-face kennenlernen.“* (EX3)

Die zuvor formulierte Hypothese könnte durch die neusten Erscheinungen, der Angst vor der plötzlichen Normalität, dem Treffen von Menschen und die damit verbundene Angst vor der Ansteckung, bekannt unter dem Cave-Syndrom (engl. Höhle), erklärt werden (Nebe 2021). Daher stellen alternative Formen, wie Netzwerkveranstaltungen mit Hilfe von AR und Holografie, eine gute Option dar. Die Interviewteilnehmer stellen sich durch AR-Netzwerken eine neue Art der Zusammenkunft und auch des Netzwerkens vor. So kann standortunabhängig miteinander kommuniziert und interagiert werden, ohne dass die Menschlichkeit verloren geht. Zudem werden Reisekosten gespart und es wird ein Anreiz für Innovationen gesetzt. AR und Netzwerken ist eine neue Form der Kommunikation und kann Neugierde wecken. Als gute Einstiegsformate stellen sich die Experten spielerische Ansätze, Produktvorstellungen oder Themen der Kollaboration oder Arbeitssicherheit vor.

*„Es eignen sich sehr gut Großkonzerne, die Geld haben, um genau sowas auszuprobieren. Die sagen ja, das ist die Zukunft und wir verändern das jetzt mit und dann machst Du das halt so: zwei Holografie-Räume und viele Aspekte zum Testen.“* (EX1)

*„Wenn wir Akquise machen, da könnte man in Richtung Gamification viele lustige Sachen machen. Dass man so eine Art Rallye, Schnitzeljagd oder sowas veranstalten, wo es verschiedene Dinge zu entdecken gibt. Also mit den HoloLens kannst Du Objekte vorab in einem Raum platzieren und jemand anderes kann dieselbe Brille aufsetzen und muss dieses Objekt zum Beispiel wiederfinden und irgendetwas damit tun.“* (EX2)

*„Wir haben auch probiert Online-Spiele wie Fortnite zu spielen […]. Das in einer AR Umgebung zu spielen, stelle ich als sehr effektiv fürs Teambuilding im Networking vor.“* (KU1)

*„Wir haben Verkehrsbetriebe als Kunden, wo es einfach viel […] um Busse, Straßenbahnen, Umbauten und dergleichen geht. Da gibt es auch ganz, ganz viele unterschiedliche Vorstellungen davon, was man damit machen könnte. Also zum Beispiel Bürger abholen, wenn irgendwelche Veränderungen anstehen, beispielweise eine neue Straßenbahnlinie. Wir sensibilisieren die Bürger schon mal, wie es dann später aussehen könnte und dann kannst Du […] die HoloLens aufsetzen und es poppt eine Straßenbahn auf, die dann später dort durchfahren wird. Also so ein bisschen das Gefühl für Räume kriegen.“* (EX2)

## Diskussion und Ausblick

Das Ziel dieser Arbeit war es, Faktoren für und gegen die Nutzung von AR und Holografie zum Netzwerken zu eruieren. Im Austausch mit den Experten und Kunden wurde deutlich, dass in der Lösungsmöglichkeit hohes Potenzial steckt und bedingt durch die COVID-19 Pandemie eine Alternative zum Traditionellen darstellt. Im Folgenden möchten wir die wichtigsten Faktoren noch einmal aufgreifen, um unsere Forschungsfrage zu beantworten.

Zusammenfassend lässt sich festhalten, dass es einige Faktoren für und gegen die Nutzung von AR und Holografie zum Netzwerken gibt, aber auch neutrale Faktoren. Eine allumfassende Darstellung der Faktoren ist in Tab. 2 zusammengefasst. Die neutralen Faktoren variieren mit jedem Menschen. Jeder Mensch hat unterschiedliche digitale Kompetenzen und so hängt es von den Vorkenntnissen ab, ob der Mensch die Möglichkeit des Netzwerkens mit AR und Holografie nutzt und akzeptiert. Gleiches gilt für das Alter der Teilnehmenden. Wie bereits in Kap. 4 diskutiert, haben die Interviewteilnehmer eine unterschiedliche Auffassung über den Einfluss des Alters auf die Nutzung und Akzeptanz von AR/Holografie Nerzwerken, weshalb wir es als neutral einstufen. Ebenso als neutral stufen wir die Gestik und Mimik ein. Sieht man die Gestik und Mimik in Echtzeit und von der realen Person (Kombination von AR und Holografie), so spricht dies für die Nutzung von AR/Holografie Netzwerken. Kommt es jedoch zu Verzögerungen und technischen Ausfällen spricht dies dagegen. Insgesamt also neutral. Dagegen sprechen jedoch auch Argumente, wie dass Netzwerken nicht ohne reale Menschen geht. Die Experten sprachen auch von einer gezwungenen Atmosphäre, wenn beispielsweise ein separater digitaler Raum zum Netzwerken eröffnet wurde. Hinzu kommen auch technische Hürden einerseits in diesen Räumen, wie das nur einer sprechen kann, und andererseits das AR und Holografie Netzwerken technisch momentan nicht umsetzbar ist und es viel technisches Equipment bedarf. Ein Gegenargument hierzu ist, dass es schon erste Probeläufe für solche neuen Veranstaltungsformate gab, beispielsweise von der 5‑HAT Digital Hub der Chemie und Gesundheit (5-HT 2021) und der GROB Werke (zreality 2020).

**Tab. 2:** Zusammenfassung der neutralen, pro und contra Faktoren zum Netzwerken mit AR und Holografie

Argumente …		
Dafür	Neutral	Dagegen
Digitale Alternative mit viel Potenzial	Alter	Keine realen Menschen
Social-Media-Kanäle als Grundlage	Kompetenzen	Gezwungene Atmosphäre
Gegenseitige Sichtbarkeit	Gestik und Mimik	Fehlende technische Umsetzbarkeit von AR und Holografie
Mehr Menschlichkeit als bei 2D-Anwendungen		Es wird viel technisches Equipment benötigt
Teil der eigenen Umgebung		
Netzwerken in kleinen Runden, die Intimität schaffen		
Alternative für Menschen mit dem Cave-Syndrom		
Finanzielle und zeitliche Vorteile		

Weiter gibt es auch Faktoren, die für das Netzwerken mit AR und Holografie sprechen. Ein Argument sind die bereits bestehenden Social-Media-Kanäle, die als Grundlage genutzt werden können, um technikaffinen Menschen in Pilotprojekten mit AR und Holografie zu vernetzen. Vorteile der AR und Holografie Kombination zum digitalen Vernetzen sind, dass die Menschen als 3D-Menschen komplett wahrgenommen werden können und so auch das Wohlfühlen gestärkt werden kann. Aufgrund der technischen Grenzen kann der informelle Teil einer Veranstaltung in kleinen Runden getestet werden, was Intimität schafft. Mit der AR und Holografie Alternative könnte auch die Übergangszeit, die Zeit bis ein sicheres Reisen und Treffen von Menschen wieder möglich ist, überbrückt werden und eine Inklusion der Menschen mit dem Cave-Syndrom geschaffen werden (Nebe 2021).

Das Hauptargument unserer Interviewteilnehmer für die Nutzung von AR und Holografie Netzwerkveranstaltungen ist die Einsparung von Zeit und Geld. An diesen Events kann von Zuhause teilgenommen werden, es bedarf keiner Reisetätigkeiten für lediglich ein paar Stunden und damit fallen viele Kosten weg, was gerade für Unternehmen ein ausschlaggebendes Argument ist.

Zuletzt werden, wie in jeder wissenschaftlichen Arbeit, die Limitationen sowie die Implikationen für Wirtschaft und Wissenschaft erörtert. Wir haben im Rahmen dieser Arbeit lediglich mit drei Experten und fünf Kunden aus dem Bereich AR und VR interviewt, weshalb hier ein Bias auftreten kann. Dies war jedoch bewusst gewählt, damit wir möglichst viele Faktoren für diese neuartige Möglichkeit des Netzwerkens analysieren konnten und auch die Barrieren durch die Expertise der Experten aufdecken konnten. Außerdem könnten zukünftige Forscher die Interviewteilnehmer der verschiedenen Branchen in verschiedene Kategorien einteilen und Unterschiede aufzeigen. Unsere Arbeit bietet sowohl für die Wissenschaft als auch Wirtschaft Implikationen. Im Wissenschaftsbereich konnten erste Vermutungen zu Nutzungs- und Akzeptanzfaktoren mit Hilfe der Interviews überprüft werden. Im Wirtschaftsbereich konnten wir feststellen, dass in der Möglichkeit des Netzwerkens mit AR und Holografie viel Potenzial und Bedarf für Lösungen stecken. Hier können Start-ups und Unternehmen innovative Geschäftsfelder gründen und die Marktlücke schließen. Wenn ein Unternehmen also nun solch ein Event zum Netzwerken testen möchte, sollten zu Beginn die Mitarbeiter spielerisch an die neue Technologie herangeführt werden. Danach sollte ein technischer Partner gesucht werden, um die Anschaffungskosten zu umgehen. Zuletzt sollte ein potenzieller Bereich aus Kapitel 4.3 gewählt werden, um zu starten. Es kann Gegenstand zukünftiger Forschungsarbeiten werden, wie sich das Netzwerken mit technologischer Hilfe weiterentwickelt und wie AR und Holografie hierbei genutzt und eingesetzt werden können.
